# Ethical relationship in the dentist-patient interaction

**DOI:** 10.4317/jced.57597

**Published:** 2021-01-01

**Authors:** Josep-Maria Ustrell-Torrent, Maria-Rosa Buxarrais-Estrada, Pere Ustrell-TorrentRiutord-Sbert

**Affiliations:** 1 MD, PhD, Full Prof. Departament of Odontostomatology. University of Barcelona. Oral Health and Masticatory System Group (IDIBELL), Spain; 2 PhD, Prof. Department of Theory and History of Education. University of Barcelona. Moral Education Research Group (GREM), Spain; 3 MD, PhD. Member of the research ethics committee of the Balearic Islands. Spain

## Abstract

**Background:**

In recent times the deontology of the dental profession has acquired special relevance as a result of problems that dentists have had to face to resolve ethical conflicts.

**Material and Methods:**

Information on deontology in the dental profession has been compiled in publications of health sciences in general and specific codes for dentistry, while expressing opinions about the experience of the authors themselves who, from a humanistic and health overview are concentrated in the dental area.

**Results:**

This article presents our point of view of how the dentist-patient relationship should be in the face of current demands from patients and society, with a focus on the crucial role of ethical issues not only in undergraduate studies but also in the consideration of ethical questions in the specialization and in the usual practice. The dental profession has a special trust in the community, and the best dentist-patient relationship should be based on that trust, honesty, providing high-quality and appropriate treatment, and keeping the patient safe and healthy.

**Conclusions:**

The patient has the right to be fully and adequately informed, as well as to participate in decisions about treatment. The dentist has a duty to put the patient first and treat her with her wishes in mind as long as these expectations are within the limits of accepted treatment. The dentist must provide dental care without discrimination or prejudice.

** Key words:**Preemptive analgesia, dental extraction, cyclooxygenases, real-time polymerase chain reaction.

## Introduction

Since civilization has been conscious of its actions, dentistry has tried to take care of an area of the human body that has a large number of functions, not only in the oral health status dimension but also in the social domain. The recognition of dentistry was difficult and only at the beginning of the last century, it was considered a university degree. It was recognized as a real specialty, and for decades, it was a part of the medical knowledge. In the last quarter of the XX century, dentistry had its own entity, but we should recognize that it depends on the general knowledge held by the physician. Therefore, everything related to dentistry should be included among the rights and duties of medicine (1) because the dentist treats patients and not only teeth.

One of the topics that we will address is the dentist–patient relationship, which requires a series of commitments to be acquired both by professionals and patients, with the obligation on both sides to respect them. The professional has the moral exigency to always tell the truth (2) and to respect confidentiality (3). In turn, the patient must be cooperative. These commitments, however, are not always met, especially when pain drowns the intelligence. According to the Jean Guitton philosopher’s thought (4), the truth does not force us to say everything we know but to say what merits to be known for justice.

## Material and Methods

Information on deontology in the dental profession has been compiled in publications of health sciences in general and specific codes for dentistry, while expressing opinions about the experience of the authors themselves who, from a humanistic and health overview are concentrated in the dental area.

## Results

This article presents our point of view of how the dentist-patient relationship should be in the face of current demands from patients and society, with a focus on the crucial role of ethical issues not only in undergraduate studies but also in the consideration of ethical questions in the specialization and in the usual practice. The dental profession has a special trust in the community, and the best dentist-patient relationship should be based on that trust, honesty, providing high-quality and appropriate treatment, and keeping the patient safe and healthy.

-Building Bridges between the Humanities and the Health Sciences

Dentistry has surpassed maturity and shares interests in health values with health sciences. In all cases, special care should be taken in the relationship with people when they seek medical care. Ustrell (5) summarized deontological concepts that have a relationship between the human being and the orofacial pathology. We should bear in mind that any condition affecting any part of the body will have an impact on the physical, psychic, and social spheres. In this respect, Soler (6) describes three axes involved in the connections between people: the vertical axis, which would represent the relationship between the health care professional and the patient; the horizontal axis, which encompasses the relationship with the community, and the personal axis. The best behavior includes a good structuring of the three axes.

Taking of actions carried out in the health area is synonymous of bioethics. Bioethics is a branch of ethics related to the systematic study of aspects related to human behavior in health sciences and health care based on moral principles and values of the human condition (7). The term bioethics was first proposed by Potter (8) in 1971 providing a new bridge point between humanities and biological sciences. The need for the emergence of bioethics becomes imperative in the face of huge scientific and technological advances in the past decades, which have not been associated with the adaptation of the human conception to this reality. When this progress is applied to the field of health sciences, the relationship between the specialist and the patient should be reformulated, since the population is increasingly more informed and more empowered of their own health (9).

In relation to the horizontal axis, Siegler (10) identified three different periods in relation to how community defines and structures the clinical relationship between physicians and patients: a) era of paternalism (from 500 b.c. to after 1960) in which the relationship is understood in a unidirectional way being the specialist, the only person responsible for decision making, with absolute trust on the part of the patient; b) era of autonomy or era of the patient – in 1969, the American Hospital Association developed the first code of the patient’s rights, which gave rise to the informed consent with provision of information being the first duty of the physicians’ principle of beneficence; and c) era of bureaucracy or era of the taxpayer in which not only the efficacy of the treatment is required but also efficiency in the management of health care resources. A final period can be added to those proposed by Siegler, in which desires of health care specialists and patients are subordinated to instructions of the administration.

On the other hand, ethical issues in medicine are less ideological than it may seem and are conceived as a dialogue between the scientific and human cultures (11). Currently, ethics is understood as the study of moral values and the way it is specified in the declaration on scientific integrity (12), aimed at protecting the physical and mental integrity of the sick people. These ethical principles include autonomy, beneficence, nonmaleficence, and justice (13). The principle of autonomy is the right of the sick person to decide on their own body, as long as he/she has the right information and without receiving any form of coercion (14). This principle is reflected in the “informed consent.” The principle of beneficence is the duty of the professional to cure the sick person. The principle of nonmaleficence (do not harm) is the obligation that one should refrain from harming others, and the principle of justice is the expression of the duty of nondiscrimination. If the adjective “dental” is added to the word ethics, we will obtain the moral conduct and the principles that govern the behaviors of the members of this profession. The clinical relationship with patients has ceased to be based on values of trust and benevolence and has become regulated by laws because health is considered as a fundamental individual right and a public good that must be protected (15).

-The Dentist–Patient Relationship

Putting now the focus of attention on the vertical axis, health care professionals require proper training according to the framework of their functions and performances, including adequate ethical training. Dentistry as a profession demands training in ethics of the patient care since care is not only dependent on professionals but also on social conditions of each situation at each particular time (16-18). The society of the 21st century is no longer the morally homogeneous society of the last century, and its values may differ from those of health care professionals (19). In this respect, Buxarrais *et al.* (18). present the briefing of the Portuguese law in which the graduate is a central Figure in the objectives of higher education, with democratic and plural spirit showing empathy and communication with the patient who seeks medical care (20). Therefore, it is especially important to promote the ethical behavior of dentistry students, with study plans that include training for acquisition of the necessary skills in this professional dimension (21,22). The professional must internalize this transformation to adapt to the social environment in which he/she lives, whether locally or globally. Competence in aptitude is very likely to be achieved with the currently established curricula, but this must be accompanied by training and the promotion of ethical reflection.

On the other hand, we should also bear in mind that it is necessary to integrate transversally the development of moral judgment and ethical decision making to act in a coherent manner. To achieve these goals, it is interesting to consider the four component model proposed by Rest and Narváez (23) based on 1) recognition and analysis of ethical problems of the profession (moral sensitivity), 2) reasoned argumentation and moral judgment of the actions to be carried out (moral reasoning), 3) commitment to the ethical principles of the profession (moral motivation), and 4) implementation of an action plan (moral action) that involves decision making. However, it is important to provide university studies with specific training (24) on ethical issues regarding the relationship between the dentist and the patient (18), the analysis and discussion of moral dilemmas that can appear in daily practice as well as ethical decision making. Although continuing education is needed for updating medical knowledge and clinical skills, the lessons learned on attitudes and ethics decision-making it is likely that they will never forget. A health-care profession demands a high level of ethical behavior toward the patient and is here where the neuralgic center of the professional practice lies.

The dentist–patient link is always related to the personal axis (6), and this link for the student means to confer importance to vocation and a feeling of love and understanding for the human nature (25). Thus, the professional practice requires honesty without concessions and above all, fidelity to a moral and ethics that place you above the miseries of human life. Moreover, medicine has been related with priesthood, in the sense of an intimate experience that others ignore and that provides satisfaction and spiritual joy. This thought reflects the relevance of our attitude, which should be as good as knowledge and skills.

-Importance of Effective Communication in Dentist–Patient Relationship

Words may or may not be therapeutic, and students must know how to use them because the dentist must be aware of disorders caused by pathology. There are examples of doctors who reported complaining about the coldness of patient care when they have been sick (26,27). Furthermore, manuals of universities with information for undergraduate and postgraduate students place special emphasis on the culture of excellence in the relationship with the patient (28). It is evident the power that the professional has, because of the condition of being so, toward the patient. For this reason, close attention should be paid to the words used, also in relation to knowledge and at the time of sharing the diagnosis. What the doctor says becomes, in many cases, an indispuTable truth for the patient (29). In addition, the different clinical decisions will be the result of a diagnosis, which may be more or less difficult, or of a possible technical difficulty inherent to treatment, but decisions should never be made due to lack of knowledge or poor technical resources availability. Therefore, our only moral obligation is to fulfill the duty guiding to maximize ethical values (30). A good healthcare professional should have the ability to observe the patient when they enter the office because the first impression can give clinical clues to a diagnosis (31).

Dentistry as a branch of medicine and as other professions of the health sciences should view the human being as a unit that integrates different parts: biological, psychological, social, cultural, and spiritual. From this holistic perspective, communication appears to be crucial in the relationship with the patient. Rogers (32) pointed out that an expert in oral health care must communicate effectively to facilitate a positive dentist–patient interaction to be able to cope, in a responsible and shared way, to the care of the patient’s health, which is more than not only the absence of disease. Thus, communication with the patient could be considered the starting point of the therapy. As stated by Watzlawick (33) everything communicates, there is no noncommunication just as there is no non conduct. Any type of behavior emits messages to our interlocutors, not only in words since gestures, facial expressions, and even silences are also forms of communication. In this respect, the health care professional should work on different aspects of verbal and nonverbal communication, as well as to develop empathy (34,35) and social skills which allow interaction with the patient. Establishing a good communication with the patient can help to reduce anxiety and possible fears in relation to the health problem or the treatment approach. This ability takes the professional beyond knowledge and skills (31). The active role of people in their health care process, freedom, and autonomy in decision making is also well known rights.

The dentist–patient communication is a key to medical praxis, especially when the person seeking medical care is elderly or of another nationality and must explain what happens to a professional who does not know and with a foreign language (36,37). The clinical history includes all information reported by the patient at the physician’s request, and an accurate diagnosis cannot be made unless dialogue has been established in a fluid and adequate manner. However, language can be studied from different fields: psychological, social, philological, linguistic, rhetorical, and anthropological, but always according to what it means for the person listening, making it difficult when communication is made through a language that is not the one that is habitually used to think, speak, or express feelings. It is important to be aware of the limitations when a person has to express what he/she wants or what he/she feels with a language that is not the language used by the listener or vice versa. In addition to the clinical history, the course of a long term treatment, which is very sensitive in the field of dentistry, can also be compromised due to a large number of loco regional anesthesia dependent treatments. In these circumstances, the dentist–patient communication is essential to provide confidence to the patient.

-A Route toward Humanism: Embracement, Security, and Confidence

The dentist should embrace patient engagement in treatment decisions providing security and confidence and reaching an agreement between the patient’s beliefs of his/her needs and what dentistry can offer (38). One of the tools that can help acquire these values is the criticism, on the part of those around us, as well as the one that comes from ourselves, self criticism. One of the principles of professional ethics is rational criticism that must always be specific, grounded, and argued to approach an objective truth (39). There is no doubt that we need others to reduce our mistakes and our ignorance, but this dialogue requires intellectual predisposition (for understanding), tolerance (to admit new ideas), and ethics training. Tolerance enables one to learn from mistakes.

Oral health, function, and esthetics are the main objectives of dentistry, but in the past years, the interest for esthetics has turned into a major reason of dentistry care since it allows the patient to achieve confidence, self esteem, and self respect (40). This requires knowledge and skills in the field of esthetic dentistry.

Furthermore, the fact that many patients come to the consultation with a lot of information from the media has probably contributed to add complexity to the dentist– patient relationship. The health care professional should know how to manage situations in relation to the patient’s wishes and expectations (14,40). There has been a growing interest in psychological aspects and subjectivity because of the emotional charge of esthetics due to the influence of culture, age, and gender (41,42). Enjoying good health, having a youthful appearance, showing a stylized body image, being sexually attractive, feeling psychologically well, achieving a high degree of personal self esteem, and being fully convinced of attaining social recognition and professional success have become primary objectives for many people. Probably, we should not give esthetics a higher importance than it really has, because the price that the patient must pay to get a good image and an attractive smile may be sometimes excessive. However, society exerts an enormous influence on everything that means fashion, triumph, and sex. We must be aware of our limitations and not fall into the excessive messages of ostentation and power. It is important to look at patients as such, and to assume responsibility, very often diluted in the team. We must return to the path of humanism43 and apply the idea of common work in our relationship. Because working as a team involves communication, and with sincerity and encouragement, the highest degree of collaboration can be achieved (44). In this respect and as responsible for integral student training, it is necessary to favor communication to create a space for favoring communication between students, classmates, and faculty members (39). This is not an easy task (4).

-Some recommendations

Finally, a series of recommendations on the necessary actions to attain dentistry professional excellence are here suggested: 1) Empathize; 2) Talk with parents or tutors; 3) Be precise in the diagnosis (it is necessary to have a deep knowledge of elementary and complementary sciences); 4) Present a logical treatment plan (a main mistake of the current approach is to base therapeutics exclusively on mechanistic or excessively technical thinking); 5) Control etiology, sTable results will be only obtained only if the cause of the problem is controlled; 6) Get sufficient clinical experience (it is not possible to learn only with theoretical courses); 7) Pursue possible and achievable results, taking responsibility for our actions; 8) Act with ethics and humanity: do good, act with moderation, know how to choose, practice the virtues, live with justice, use reason, use your heart, be friendly, and be happy (45); 9) Learn from our mistakes; and 10) learn from our teachers.

The relationship between the patient and the dentist should be adapted to the new times as well as to the changes and difficulties associated with patients presenting with confused or fragmentary knowledge. Misunderstandings should be clarified providing appropriate and clear information to reinforce the dentist–patient interaction link, which will contribute to improve patient’s oral health. We should listen, reassure, diagnose, reflect, dialogue, and treat so that in this way, patients will obtain the greatest benefit from the dentist–patient interaction. It is necessary to keep in mind the importance of an adequate clinical history, physical examination, and complementary studies, and that we must observe and reflect before acting. Besides being competent in our profession, the patient should be always in the first place and that we are morally obliged to protect him (46), to counteract the deleterious effect of the lack of time on the dentist–patient relationship. It is urgent to reestablish and reinforce the essential role the dentist–patient interaction in the medical performance, combining technical advances with the humanistic part of our profession (47).

## Conclusions

The patient has the right to be fully and adequately informed, as well as to participate in decisions about treatment. The dentist has a duty to put the patient first and treat her with her wishes in mind as long as these expectations are within the limits of accepted treatment. The dentist must provide dental care without discrimination or prejudice.

## References

[1] Martínez P (2014). The doctor patient relationship to Unesco as an Intangible heritage of humanity. https://www.cgcom.es/sites/default/files/thedoctor_patient_relationship//files/assets/common/downloads/publication.pdf?uni=3036561d18712e359d44a1ed9154a572..

[2] Martínez Montauri J (2012). New paradigms in the doctor patient relationship.

[3] Silva Junior DN, Lima de Araújo J, Gurgel Cosme do Nascimento E (2017). Privacidade e confidencialidade no contexto mundial de saúde: Uma revisão integrativa. Rev Bio Der.

[4] Guitton J (2004). Lo que yo creo. Razones por las que creer.

[5] Ustrell JM (2017). Relación odontólogo paciente. Odontólogos de Hoy.

[6] Soler JM (2011). La plaça de Diognet.

[7] Ustrell JM, Borges RD (2010). Ética y deontología profesionales.

[8] Potter VR (1971). Bioethics: bridge to the future.

[9] Celedón C (2016). Relación médico paciente. Rev Otorrinolaringol Cir Cabeza Cuello.

[10] Siegler MA (1997). The contribution of clinical ethics to patient care. In: Forum trends in experimental and clinical medicine.

[11] Aragay I (2020). http://www.ara.cat/cronica/NURIATERRIBAS-problemesmedicinaideologicssembla_0_661133933.html.

[12] Casado M, Patrão Neves MC, de Lecuona I, Carvalho AS, Araújo J (2016). Declaració sobre integritat científica.

[13] Beauchamp TL, Childress JF (1999). Principios de ética biomèdica.

[14] Patrick AC (2014). The dentist patient relationship: re modelling autonomy for dentistry.

[15] Morlans M (2016). El conocimiento científico y el saber de la ética: el paradigma de la medicina clínica. Folia Humanística.

[16] Buxarrais MR (1997). La formación del profesorado en educación en valores.

[17] Buxarrais MR (2016). El cuidado ético como camino hacia el ser. In: Buxarrais MR, Burget M, editors. Aprender a ser. Por una pedagogía de la interioridad.

[18] Buxarrais MR, Azevedo M da C (2017). Competencias y competencia ética en la educación superior. In: Vila ES, et al., editors. Competencias éticas y deontología profesional en la universidad.

[19] Solsona JF (2016). El paciente informado. Los conflictos éticos más frecuentes relacionados con la asistencia sanitaria.

[20] Kemparaj VM, Panchmal GS, Jayakumar HL, Kadalur UG (2016). Qualitative assessment of ethical issues in dental practice: an expert opinion. J Educ Ethics Dent.

[21] Lantz MS, Bebeau MJ, Zarkowski P (2011). The status of ethics teaching and learning in U.S. dental schools. J Dent Educ.

[22] Narasimhan M (2015). Dental ethics education. A responsibility of an academician. J Educ Ethics Dent.

[23] Rest J, Narváez D (1994). Moral development in the professions. Psychology and applied ethics.

[24] Lucietto DA, Amancio Filho A, Vasconcellos MM (2016). Formação de estudantes de odontologia em tempos de SUS. Revint.

[25] Ustrell JM (2010). Dos Mestres de la meva vida.

[26] Ustrell JM (2010). Dos Mestres de la meva vida.

[27] Rosenbaum EE (1988). A taste of my own medicine: when the doctor is the patient.

[28] Arilla A, Serra O (2014). Manual d'acollida a l'alumnat. L'Hospitalet: Fundació Josep Finestres.

[29] Jacquot J (2005). Trust in the dentist patient relationship. A review. J Young Investig.

[30] Gracia D (2016). El paciente Informado.

[31] Vilardell M (2009). Ser metge. L'art i l'ofici de curar.

[32] Rogers C (1989). The Carl Rogers reader.

[33] Watzlawick P (1981). Teoría de la comunicación humana.

[34] Hoffman ML (2000). Empathy and moral development. Implications for caring and justice.

[35] Nash DA (2007). On ethics in the profession of dentistry and dental education. Eur J Dent Educ.

[36] Yamalik N (2005). Dentist patient relationship and quality care 3. Communication. Int Dent J.

[37] Riutord P (2010). Salut i comunicació. Diari de Balears. Diari de Balears.

[38] Garbin CA, Garbin AJ, Saliba NA, de Lima DC, de Macedo AP (2008). Analysis of the ethical aspects of professional confidentiality in dental practice. J Appl Oral Sci.

[39] Popper KR (1997). En busca de un mundo mejor.

[40] Riutord P (2010). La bellesa d'un somriure.

[41] Ustrell JM, Ustrell G (2010). Aspectos subjetivos de la estética facial. In: Ustrell Torrent JM, editor. Manual de Ortodoncia.

[42] Braga SA, Abrão J, Capelozza L, de Assis CA (2006). Análise facial subjetiva. R Dent Press Ortodon Ortop Fac.

[43] Loignon C, Allison P, Landry A, Richard L, Brodeur JM, Bedos C (2010). Providing humanistic care: Dentists' experiences in deprived areas. J Dent Res.

[44] Jaspers K (2013). La Idea de la universidad.

[45] Chalita G (2003). Os dez mandamentos da ética.

[46] Guimarães M (2017). A falta de tempo está a destruir a relação médico doente. Rev Ordem Médicos.

[47] Rodrigues R (2017). A dimensão ética na formação dos cirurgões dentistas no estado de Minas Gerais - Brasil.

